# The Role of C-Type Lectin Receptor Signaling in the Intestinal Microbiota-Inflammation-Cancer Axis

**DOI:** 10.3389/fimmu.2022.894445

**Published:** 2022-05-10

**Authors:** Muhan Li, Runfeng Zhang, Ji Li, Jingnan Li

**Affiliations:** ^1^ Department of Gastroenterology, Peking Union Medical College Hospital, Chinese Academy of Medical Sciences and Peking Union Medical College, Beijing, China; ^2^ Key Laboratory of Gut Microbiota Translational Medicine Research, Peking Union Medical College Hospital, Chinese Academy of Medical Sciences and Peking Union Medical College, Beijing, China

**Keywords:** C-type lectin receptor, pathogen-associated molecular patterns, damage-associated molecular patterns, inflammatory bowel disease, colitis associated cancer

## Abstract

As a subset of pattern recognition receptors (PRRs), C-type lectin-like receptors (CLRs) are mainly expressed by myeloid cells as both transmembrane and soluble forms. CLRs recognize not only pathogen associated molecular patterns (PAMPs), but also damage-associated molecular patterns (DAMPs) to promote innate immune responses and affect adaptive immune responses. Upon engagement by PAMPs or DAMPs, CLR signaling initiates various biological activities *in vivo*, such as cytokine secretion and immune cell recruitment. Recently, several CLRs have been implicated as contributory to the pathogenesis of intestinal inflammation, which represents a prominent risk factor for colorectal cancer (CRC). CLRs function as an interface among microbiota, intestinal epithelial barrier and immune system, so we firstly discussed the relationship between dysbiosis caused by microbiota alteration and inflammatory bowel disease (IBD), then focused on the role of CLRs signaling in pathogenesis of IBD (including Mincle, Dectin-3, Dectin-1, DCIR, DC-SIGN, LOX-1 and their downstream CARD9). Given that CLRs mediate intricate inflammatory signals and inflammation plays a significant role in tumorigenesis, we finally highlight the specific effects of CLRs on CRC, especially colitis-associated cancer (CAC), hoping to open new horizons on pathogenesis and therapeutics of IBD and CAC.

## Introduction

IBD consists of Crohn’s disease (CD) and ulcerative colitis (UC). IBD majorly affects young adults, which substantially alters their life quality and causes enormous financial burden for health care (1, 2). The incidence of IBD in western countries has been high for decades, with over 1 million patients in the USA and 2.5 million in Europe (1). Many newly industrialized countries around the world also have a rapidly increasing incidence in recent years (1). Although the exact pathogenesis of IBD remains obscure, numerous evidence supports that aberrant innate and adaptive immune response against the intestinal pathogenic and commensal microbiota are responsible for IBD in genetically susceptible populations (3).

Fungus, as an indispensable component of intestinal microbiota, take a crucial part in maintaining the gut homeostasis, and its dysbiosis might contribute to the pathogenesis of IBD (4, 5). There are trillions of microorganisms resided in human gut, including bacteria, fungi, archaea and viruses (4), and metagenomic sequencing reveals that their collective genome are 100 times more than our own genome (6, 7). Over 99% of these genes belong to bacteria, and over 90% of intestinal bacteria belong to two phyla, *Firmicutes* and *Bacteroidetes (* 7, 8). Whereas fungi only makes up 0.1% of the total human gut microorganisms, among which the phyla *Ascomycota*, *Basidiomycota* and *Chytridiomycota* are predominant (4). Bacteria have long been the focus of intestinal flora research, while the role of fungi have been undervalued due to their low proportion and relatively low infection rate. In recent years, the number of immunocompromised patients has largely expanded due to increasing prevalence of HIV and the wide use of chemotherapy, organ transplantation, and immunosuppressive agents. Opportunistic infections and mortality caused by fungi have attracted more and more attention, thus understanding immune responses against fungi is of great immediate significance. Now people have realized that both bacteria and fungi exert important functions in maintaining the intestinal homeostasis, and their dysbiosis have been indicated to take part in multiple diseases, including IBD (4, 9, 10).

The recognition of fungi are predominantly dependent on the C-type lectin receptor (CLR) family, an important family of pattern recognition receptors (11). The basic theory of the interplay between fungi and CLRs is the Janeway’s theory that antigen presenting cells (APCs) recognize and respond to non-self molecules through PRRs binding to quite conserved pathogen-associated molecular patterns (PAMPs) (12). Upon ligation with conserved PAMPs expressed on a wide variety of fungal cell walls, CLRs initiate intricate downstream signal network and ultimately induce corresponding innate and adaptive immune responses. Distinct fungi have different cell wall composition thus expressing different PAMPs (13). The combination of many PAMPs is just like a fingerprint, which triggers the activation of specific signaling pathways to distinguish between different microorganisms and fine-tune the immune response against specific microorganism (13, 14). Although CLRs are at the very core of intestinal fungal immunity, it is unreasonable to ignore the crosstalk and collaboration between CLRs and other subsets of the PRR family, including well-studied Toll-like receptors (TLRs), inflammasome-related nucleotide-binding oligomerization domain (NOD)-like receptors (NLRs), and RNA-detecting retinoic acid-inducible gene-I(RIG-I)-like receptors (RLRs) (14, 15). Much more diverse immune responses can be induced by engaging several PRRs simultaneously to ensure the effectiveness of immune response (14).

However, PAMPs are still insufficient to explain many phenomena, such as tumor immunity, transplant rejection and autoimmunity. Matzinger’s danger model supposes that APCs also recognize and respond to endogenous damage-associated molecular patterns(DAMP) derived from damaged or distressed tissues, which nicely complement Janeway’s self-non-self model (16). The danger model is supported by mounting evidences. For example, many CLRs have been demonstrated to bind not only PAMP, but also DAMP, with different ligation to produce different responses. By recognizing DAMPs that are released by distressed tissues or expressed during malignant transformation, CLRs have been proven to participate in the pathogenesis of multiple inflammatory disorders and malignant tumors, with different CLRs playing different or even opposite roles (17–21).

In order to comprehensively understand the significance of CLRs family in the interplay among intestinal microbiota-inflammation-cancer axis, we selected several typical representatives from each of the four CLRs categories, including Mincle, Dectin-3, Dectin-1, DCIR, LOX-1, and DC-SIGN. In addition, there is a consistent core downstream molecule CARD9 in the downstream signaling pathways of various CLRs, therefore CARD9 also takes a central part in intestinal immunity. In this review, we summarized the cutting-edge research progress of these molecules in the field of intestinal inflammation and CRC, which is of great significance for understanding the pathogenesis of IBD and colitis associated cancer (CAC) and developing new therapeutic strategies.

## The C-Type Lectin Receptor Family and Its Subtype

Lectins can selectively bind to specific carbohydrate structures, which are extremely useful tools for understanding the information encoded in carbohydrates, studying the characterization of polysaccharides and glycoproteins, recognizing cell-molecule and cell-cell interactions, especially investigating the changes in malignancy (22, 23). In 1988, Drickamer organized numerous lectins into several categories, two of which are Ca^2+^-dependent C-type and thiol-dependent S-type (24). The C-type lectin family has boomed since then, with a thousand identified members from diverse animal species (25). Through characterizing the structures and functions of different domains, their carbohydrate-binding activities were demonstrated to be mediated by special carbohydrate recognition domain (CRD), whose compact globular structure was not similar to any known protein fold (25).

The C-type lectin receptors have at least one C-type lectin-like domains (CTLD) in their extracellular region (C-terminus), which are homologous to CRD and responsible for recognizing specific carbohydrate structures mainly in a Ca^2+^-dependent manner (14, 25). CLRs whose CRD contains Glu-Pro-Asn (EPN) tripeptide motifs could bind mannose, glucose, fucose and N-acetylglucosamine (GlcNAc), whereas CLRs whose CRD contains another tripeptide motif, Glu-Pro-Asp (QPD), bind galactose and N-acetylgalactosamine (GalNAc) (26). In fact, CLRs do not always bind carbohydrate structures or depend on Ca^2+^ (14, 25), for example, proteins and lipids can also act as ligands of CLRs (27). Most CLRs are transmembrane receptors, but some can be released as soluble proteins, such as mannose-binding-lectin (MBL) (28). A lot of CLRs were initially identified to be expressed on dendritic cells (DCs) including the well-known Dectin-1and DC-SIGN (29–31). Following studies found that monocytes, macrophages, B cells, neutrophils, and intestinal epithelial cell (IEC) may also express certain CLRs (14, 32). Although CLRs are classically considered to be capable of recognizing carbohydrate structures in fungal cell wall, many evidences suggest that they are also involved in sensing a large range of pathogens including bacteria, viruses, and helminths, as well as DAMPs (14, 18, 33–38). Upon ligation, distinct CLRs can variably affect endocytic, phagocytic, proinflammatory or anti-inflammatory responses, which is determined by the extremely versatile CLR signaling system (27). Finally, CLRs connect innate immunity with adaptive immunity through antigen internalization, antigen presentation and T cell activation (27, 34, 39, 40).

CLRs can be roughly divided into four groups with different cytoplasmic signaling motifs (**Table 1**) (27, 41). Immunoreceptor tyrosine-based activating motif (ITAM)-coupled CLRs either have an evident ITAM motifs consisting of YxxL tandem repeats in their cytoplastic tail with Y representing tyrosine, or interacts with ITAM-containing adaptors. Fc receptor γ chain (FcRγ) is the most common ITAM-containing adaptor proteins for CLR-mediated downstream signal transduction, such as Mincle (18). Hemi-ITAM-(hemITAM)-bearing CLRs only contain a single tyrosine (Y) within their cytoplasmic YxxL motif, such as Dectin-1 (27, 42). After CLRs binding to ligands, tyrosine(s) within the ITAM or hemITAM are phosphorylated to recruit spleen tyrosine kinase (Syk) and subsequently assemble the CARD9/Bcl-10/MALT1 complex, thus leading to NF-κB activation, which affects many aspects of both innate and adaptive immunity (43, 44). Therefore, these ITAM or hemITAM CLRs generally function as activating receptors to trigger and potentiate immune responses, although they may also play an inhibiting role in some cases (45–48). Unlike ITAM or hemITAM-containing CLRs mentioned above, immunoreceptor tyrosine-based inhibitory motif (ITIM)-containing CLRs, such as DCIR, usually negatively regulate the signaling pathway of other PRRs, which is achieved by recruiting tyrosine phosphatases SHP-1 or SHP-2 instead of tyrosine kinase (27). ITAM-ITIM independent CLRs, such as human DC-SIGN and LOX-1, lack typical ITAM or ITIM signaling motifs (41).

**Table 1:** C-type lectin receptors and its immune recognition.

	Signal module	Other names	Expression	PAMP	DAMP
Dectin-1	hemITAM	CLECSF12, CLEC7A	Mo, Mϕ, DC, Neu, Eos, B cell, T cell, bronchial epithelial cell, intestinal epithelial cell	β-1, 3-glucan	Vimentin, carbohydrate on tumor
Mincle	ITAM	CLECSF9,CLEC4E	activated Mϕ, DC, Neu, B cell	α-mannan, various glycolipids, (TDM)	SAP130, cholesterol sulfate, cholesterol crystal, β-GlcCer
Dectin-3	ITAM	MCL, CLECSF8, Clec4d	Mϕ, Mo, Neu, DC	α-mannan, TDM	——
DCIR	ITIM	CLECSF6, Clec4a2	DCs, Mo, Mϕ, B cell, granulocyte, activated T cell	fucose, mannose	carbohydrate on tumor
DC-SIGN	ITAM-ITIM independent	CD209 murine homolog: SIGN-R1, SIGN-R3	Human DC, Mϕ	fucose, mannose	ICAM-3, ICAM-2, Mac-1, Mac-2BP, MSPL/TMPRSS13, CEA, CEACAM1, carbohydrate on tumor
LOX-1	ITAM-ITIM independent	OLR1, Clec8a	endothelial cell, smooth muscle cell, cardiomyocyte, adipocyte, platelet, Mo, Mϕ, DC, B cell, chondrocyte, intestinal cell	G+ bacteria, G- bacteria	Ox-LDL, Ox-HDL, HSP, PS, apoptotic bodies, AGEs, platelets

ITAM, immunoreceptor tyrosine-based activating motif; hemITAM, hemi-ITAM; ITIM, immunoreceptor tyrosine-based inhibitory motif; Mϕ, macrophage; DC, dendritic cell; Neu, neutrophil; Mo, monocyte; Eos, eosinophil; TDM, trehalose-6,6’-dimycolate; G+, Gram-positive; G-, Gram-negative; SAP130, spliceosome-associated protein 130; β-GlcCer, β-glucosylceramides; ICAM-3, intercellular adhesion molecule-3; ICAM-2, intercellular adhesion molecule-2; Mac-2BP, Mac-2-binding protein; MSPL, mosaic serine protease large-form; TMPRSS13, transmembrane protease serine 13; CEA, carcinoembryonic antigen; CEACAM1, carcinoembryonic-antigen-related cell-adhesion molecule-1; Ox-LDL, oxidized low density lipoprotein; Ox-HDL, oxidized high density lipoprotein; HSP, heat shock protein; PS, phosphatidylserine; AGEs, advanced glycation end-products.

It should be noted that although the intracellular structural motifs of CLRs are crucial to the molecular signaling pathways, they are still not enough to accurately characterize or predict downstream signaling transduction and following immune responses (41). Motif context, receptor location, multimerization of CLRs, ligand type and concentration, crosstalk with other PRR all contribute to the flexibility of signaling pathways (41).

## The Role of CLRs in the Interplay Between Fungal Microbiota and IBD

The intestinal bacterial microbiota is skewed in IBDs, whose dysbiosis has been thoroughly investigated and proven a substantial significance in IBD pathogenesis (49, 50). While the fungal microbiota is relatively poorly studied. In recent years, mounting clues suggest that fungi dysbiosis also take part in IBD pathogenesis. An early preliminary study showed changes in the intestinal fungal diversity and composition of IBD patients (51). Compared to the noninflamed mucosa, inflamed mucosa shows elevated fungal richness and diversity, which is characterized by expanded opportunistic pathogenic fungi including *Candida* spp., *Gibberella moniliformis* and *Cryptococcus neoformans (* 52). Subsequently, many studies consistently found that *Candida* spp. (e.g., *Candida tropicalis*) showed significantly increased abundance in IBD patients, suggesting its involvement in the pathogenesis of IBD (53–55). More recently, fungal composition and diversity in the feces of IBD patients and healthy subjects was identified and compared by ITS2 sequencing, and results showed fungal dysbiosis in IBD, with decreased *Saccharomyces cerevisiae*, increased *Candida albicans* and increased *Basidiomycota*/*Ascomycota* ratio (56). Interestingly, genotype–fungal microbiota analysis implied that genes could influence the intestinal fungal dysbiosis in IBD (56). A latest multi-omics study revealed fungi-related and bacteria-related metabolomics profiles in CD patients charactered by increased amino acid degradation and specific fecal volatile organic compounds (VOCs) (57). Murine colitis models, as a counterpart to human IBD, also showed alterations in intestinal fungal communities (58). Compared with normal controls, dextran sulfate sodium (DSS)-induced colitis mice showed increased *Candida*, *Penicillium*, *Wickerhamomyces*, *Alternaria* whereas decreased *Cryptococcus*, *Phialemonium*, *Wallemia* (58).

Intestinal fungi play a dual role in intestinal inflammation. The most commonly observed intestinal commensal fungal species *Candida albicans* has been shown to contribute to the development of IBD. *Candida albicans* could induce anti-*Saccharomyces cerevisiae* antibodies (ASCA), a biomarker of CD (59, 60). Other researchers then showed that gut inflammation greatly promoted *Candida albicans* colonization in mice, and *C. albicans* exacerbated DSS-colitis in turn (61). In fact, *Candida* could delay healing in trinitrobenzene sulphonic acid (TNBS)-induced mouse colitis model, and antifungal fluconazole therapy or probiotic lacidofil treatment during *Candida* infection could help the restoration of colonic damage during colitis (62). Besides, in chronic recurrent DSS-colitis, intestinal fungi can also harmfully translocate into abnormal sites (e.g., colonic mucosa, mesenteric lymph nodes and spleen) and aggravate disease (58). Intriguingly, gut fungal dysbiosis also had persistent effects on local and peripheral immunity and lead to exacerbated allergic airway disease (63), later study demonstrated the mechanism was mediated by CX3CR1^+^ mononuclear phagocytes (64).

However, fungi also exhibit a protective function. For example, *Saccharomyces boulardii* acts as a probiotic yeast to suppress intestinal colonization by *Candida albicans*, suppress intestinal inflammation and facilitate mucosal restoration by regulating VEGFR signaling and angiogenesis (65, 66). What’s more, fungi can interact with intestinal bacteria to ensure a balance between fungi and bacteria. Compared with normal control, the depletion of fungi lead to aggravated DSS-induced mouse colitis, which is associated with substantial alterations in mucosal bacterial composition, for example, *Bacteroides* and *Lactobacillus* increased while butyrate-producing *Clostridia* XIVa and *Anaerostipes* reduced (58). Some strains from clusters IV, XIVa and XVIII of *Clostridia* are benefical to the host, and oral administration of them can ameliorate mice colitis, because these bacteria lack prominent virulence and have been proven to provide a TGF-β-rich environment to promote the growth and differentiation of anti-inflammatory cells and the upregulation of anti-inflammatory molecules, especially regulatory T cells (Treg) and IL-10 (67). Interestingly, commensal bacteria depletion leads to susceptibility to colitis, and intestinal mono-colonization with single species of fungi (*Candida albicans* or *Saccharomyces cerevisiae*) can overturn this susceptibility (68). That means commensal fungi can exhibit equivalent protective benefits just as the commensal bacteria do, through providing tonic microbial stimulation, protecting against mucosal injury and elaborately tuning the responsiveness of circulating immune cells (68). Persistent fungal intestinal colonization and the mannans structure of fungal cell wall are required for the beneficial role of commensal fungi (68). The protective effects of commensal fungi may partly explain why antifungal treatment exacerbates colitis in wild-type mice (63).

**Figure 1:**
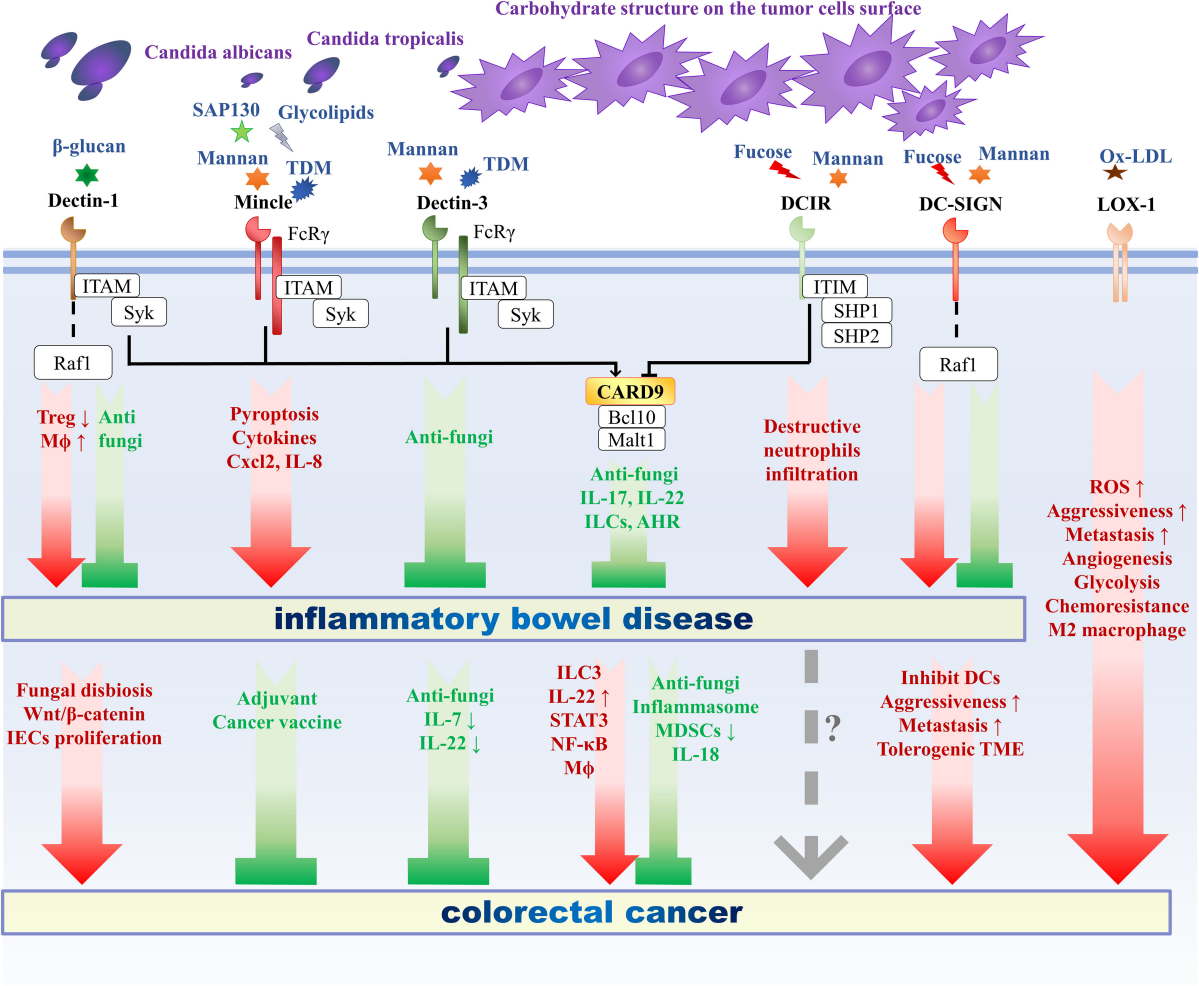
The role of C-type lectin receptors in intestinal inflammation and oncogenesis. The role of C-type lectin receptors in intestinal inflammation and oncogenesis. CLRs include different categories, among which Mincle and Dectin-3 belong to ITAM-coupled CLRs, Dectin-1 belongs to hemITAM-bearing CLRs, DCIR belongs to ITIM-containing CLRs, and DC-SIGN and LOX-1 belong to ITAM-ITIM independent CLRs. CARD9 functions as a crucial adaptor molecule downstream of various CLRs. Upon ligation with ligands derived from fungi or cancer, different CLRs initiate different signal transduction pathway and following immune responses, thus exerts different roles in the pathogenesis of inflammatory bowel disease and colorectal cancer. Mincle and DCIR exacerbate IBD, Dectin-3 and CARD9 prevent IBD, while Dectin-1 and DC-SIGN can both exacerbate and ameliorate IBD. As for CRC, Mincle and Dectin-3 play a protective role against tumor progression, Dectin-1, DC-SIGN and LOX-1 deteriorate CRC, CARD9 plays a dual role, and the role of DCIR in CRC remains unclear. CLRs, C-type lectin receptors; ITAM, immunoreceptor tyrosine-based activating motif; hemITAM, hemi-ITAM; ITIM, immunoreceptor tyrosine-based inhibitory motif; TME, tumor microenvironment; AHR, aryl hydrocarbon receptor; Mϕ, macrophage; ROS, reactive oxygen species; DC, dendritic cell; MDSC, myeloid-derived suppressor cell; IEC, intestinal epithelial cell; ILC, innate lymphoid cell; Treg, regulatory T cell.

As discussed above, CLRs function as the most important PRRs that regulate antifungal immunity. The maintenance of intestinal homeostasis requires the role of CLRs, thus responding to the pathogenic microorganisms and tolerating the normal intestinal commensal flora. The following will introduce the effects of several classic CLRs signaling pathways on intestinal immunity and IBD (**Figure 1**).

### Dectin-1

As one of the most well-researched CLRs, Dendritic cell-associated C-type lectin-1 (Dectin-1) is a membrane protein, consisting of a hemITAM-containing cytoplasmic tail, transmembrane region, a stalk and extracellular CTLD (27, 29). Originally identified as a dendritic cell receptor in the mouse through subtractive cDNA cloning (29), Dectin-1 is now known to be present on various kinds of cells in human and mice, including monocytes, macrophages, neutrophils, eosinophils (69), B cells (69), some subgroups of T cells (69), bronchial epithelial cells (70), and intestinal epithelial cells (32), demonstrating that Dectin-1 is not a myeloid restricted CLRs (**Table 1**).

Atypically, Dectin-1 recognize carbohydrate in a Ca^2+^-independent manner (71). Dectin-1 can recognize β-1, 3-glucans derived from diverse sources, including plants, bacteria and nearly all fungi (e.g., *Pneumocystis carinii*, *Candida albicans*, and *Aspergillus fumigatus*) (71–75). Particulate but not soluble β-glucans can activate Dectin-1 through forming Dectin-1-clustered synapse-like structures and excluding tyrosine phosphatases at β-glucan contact sites (76). Then Dectin-1 mediates signaling pathways through Syk, CARD9/Bcl-10/MALT1 complex and sometimes noncanonical caspase-8, to induce phagocytosis, production of reactive oxygen species (ROS) and maturation of IL-1β, which is important for protective immune responses (76, 77). In addition to be coupled with Syk, Dectin-1 can induce helper T cell (Th cell) differentiation and immune responses through another independent signaling pathways *via* serine-threonine kinase Raf1 (78). Besides, Dectin-1 also have endogenous ligand vimentin, which contributes to the pathogenesis of atherosclerosis (79).

Dectin-1 plays pleiotropic functions in the intestinal mucosa barrier to defense against pathogens invasion. Intestinal epithelial cells (IECs) provide the biggest contact area to interact with luminal abundant antigens, and has been proven to express the β-glucan receptor Dectin-1 and other downstream adaptor proteins (32). Based on Dectin-1 dependent pathway, human IECs respond to intestinal β-glucans stimulation through secretion of multiple cytokines and chemokines (e.g., IL-8 and CCL2), emphasizing the significance of CLRs-mediated interactions between fungi and non-myeloid cells such as IEC (32). In addition to mechanical defense function, intestinal mucosa also contains many types of biologically active substances, such as mucin glycoproteins, secreted immunoglobulins (sIgA and IgG), which is also related to Dectin-1. Besides physically separating intestinal epithelia and the luminal microbiota or food to protect mucosa barrier, the dense mucus layer also prevents intestinal inflammation and enhances oral tolerance by mucin MUC2-mediated formation of galectin-3 (Gal-3) -Dectin-1-FcγRIIB complex, which inhibits NF-κB activation, downregulates pro-inflammatory cytokine and imprints DCs with anti-inflammatory characters (80). The molecular basis is that the N-glycan structures of Dectin-1 as well as SIGN-R1 can be recognized by Gal-3 (81). Another study found that Dectin-1 that is expressed on microfold cells also mediate the intestinal reverse transcytosis of secretory IgA (SIgA) to transport from lumen to the gut-associated lymphoid tissue (GALT) (82).

Though it has not been fully studied, some researchers have recognized that Dectin-1 is also involved in adaptive immunity in the gastrointestinal tract. Both *in vitro* and *in vivo*, Dectin-1-Syk-CARD9 signaling has been implicated to connect the innate immunity and adaptive immunity *via* promoting the maturation of DCs, the secretion of proinflammatory IL-6, TNF, IL-23, and the differentiation of IL-17-producing Th-17 cells (83). It is noteworthy that Dectin-1-induced protective Th17 responses are critical in the human mucosal antifungal defense responses, for example distinguishing between colonizing and invasive *Candida* based on their morphological transition from yeast to hyphae (84). However, uncontrolled Th17 activation and polarization also contribute to pathogenic inflammatory conditions, such as IBD and colitis-associated cancer (85). In addition, Dectin-1 is required for driving intestinal fungal-specific CD4^+^ T-cell immune responses during fungal infection in mice, and the deficiency of Dectin-1 leads to substantially aberrated CD4^+^ T responses in the mesenteric lymph nodes with increased T cell apoptosis and defective T cell activation (86).

Dectin-1 exerts protective function, whose deficiency strongly influence susceptibility to intestinal inflammation. An early classic study demonstrated that Dectin-1-deficient mice showed more susceptibility to colitis when treated with DSS, as a result of impaired immune responses against specific commensal fungi companied by increased opportunistic pathogenic fungi such as *Candida* and *Trichosporon*, whereas decreased nonpathogenic *Saccharomyces (* 87). After supplementing with an opportunistic pathogen *Candida tropicalis*, Dectin-1-deficient mice exhibited more aggravated colitis and increased pro-inflammatory molecules (such as IFN-γ, IL-17, IL-23p19, TNF-α) in contrast to wild-type mice, but supplementing with nonpathogenic fungus *Saccharomycopsis fibuligera* did not contribute to mouse colitis (87). Inhibiting fungal dysbiosis with antifungal treatment (fluconazole) could ameliorate colitis in Dectin-1-deficient mice (87). Interestingly, the altered gastrointestinal and vaginal mucosal antifungal immunity induced by Dectin-1 deficiency is also affected by the genetic background of the host, because of different canonical or noncanonical signaling pathways downstream of Dectin-1 in distinct mouse strains (C57BL/6 and BALB/c) leading to diverse immune responses (88). In humans, there is evidence that Dectin-1 expression is upregulated on various immune cells that take part in the intestinal inflammation, such as macrophages and neutrophils, which results in the overexpression of Dectin-1 in inflamed colon tissues in both IBD and diverticulitis patients (89). And the polymorphism of human Dectin-1 gene is strongly correlated to medically refractory UC (87). Besides, defective expression and function of Dectin-1 on monocyte also promote systemic lupus erythematosus, rheumatoid arthritis and many inflammatory diseases (90).

Intriguingly, Dectin-1 actually serves as a double-edged sword. In mouse arthritis model, the stimulation of Dectin-1 by β-glucan causes inflammatory cells and cytokines responses and is harmful to the host (91). What is more, because Dectin-1-induced anti-microbial peptides could inhibit *Lactobacillus murinus* growth, the deficiency of Dectin-1 can elevate the abundance of *Lactobacilli* in the mice gut, which promote regulatory T cell (Treg) development and consequently suppress the development of colitis induced by DSS or CD45RB^high^CD4^+^ T cell (92). Besides, there is a similar pattern existing in humans. *Lactobacillus salivarius*, which is closely related to *L. murinus*, has been demonstrated to decrease in the intestinal flora of IBD patients, and *L. salivarius* also induce the upregulation of anti-inflammatory TGF-β and IL-10 in intestinal immune cells just like the *L. murinus* do (92). These observations point out the important role of Dectin-1 in balancing intestinal proinflammatory immunity and anti-inflammatory immunity through adjustment of commensal microbiota. A latest study also found that experimental colitis was attenuated in mice with Dectin-1-deficient myeloid cells, while the deficiency of mannose receptor (MR) exacerbates colitis (93). Through the elevated production of chemokine CCL2, Dectin-1 promotes the enrichment of Ly6C^high^CCR2^high^ monocyte subset in the blood and their infiltration to the inflamed colon tissue, thus contributing to the proinflammatory profile of intestinal macrophages during colitis (93). Furthermore, both overexpression of Dectin-1 and reduction of MR expression are associated to colonic inflammation in IBD biopsy specimens (93).

In addition to intestinal microbiota, the ingredients in daily foods may contain ligands for Dectin-1, which also affects intestinal inflammation. Because of the Dectin-1/IL-17F/anti-microbial peptides (calprotectin) axis, β-glucans components from food can modulate the expansion of commensal bacterium *L. murinus* to influence the Treg expansion in mice (94). Therefore, β-glucan-free diet alleviates the mouse colitis, with significantly decreased infiltration of inflammatory cells in colonic epithelia or lamina propria compared to normal diet (94). While laminarin, a Dectin-1 antagonist derived from brown algae kombu, have been proven to ameliorate the mouse DSS-colitis through intestinal *L. murinus* expansion and Treg accumulation (92). Interestingly, the effects of Dectin-1 antagonist in experimental mouse colitis are similar to other observations of Dectin-1-deficiency and β-glucan-free food (92, 94). Therefore, these studies indicate the important role of diet and Dectin-1 in intestinal inflammation, which inspire a new idea to prevent IBD by developing beneficial foods.

Both excessive Dectin-1 inhibition and excessive Dectin-1 activation lead to aggravated colitis because of uncontrolled fungal invasion or the suppression of anti-inflammation responses respectively (92). Overall, a delicate balance between agonist and antagonist for Dectin-1 is important for intestinal homeostasis.

### Mincle

After exposure to various stimuli, macrophage inducible C-type lectin (Mincle) is mainly upregulated on macrophages (18). Mincle commonly acts as an activating receptor that couples with the ITAM-containing FcRγ, and initiates a downstream signaling pathway through Syk recruitment, CARD9/Bcl-10/MALT1 complex formation, and NF-κB activation to induce immune responses including the production of cytokines and chemokines and the recruitment of inflammatory cells (18, 41, 95).

Mincle possesses the capacity to recognize plenty of PAMP and DAMP (**Table 1**) (95). PAMP, such as α-mannan and various glycolipid, can be derived from versatile microbial pathogens and commensals. For example, glycolipid in mycobacterial cell wall whose chemical structure is trehalose-6,6’-dimycolate (TDM), can be recognized by Mincle (96). After ligation, TDM can induce the upregulation of Mincle (97). Mincle is essential for TDM-induced immune responses, such as inflammatory cytokines production and granuloma formation (96). DAMP include cholesterol sulfate (19), cholesterol crystals (20), β-glucosylceramides (β-GlcCer) (98), and spliceosome-associated protein 130 (SAP130) (18), which is released upon cell death.

The role of Mincle in mucosal barrier function and intestinal immunity has only been revealed in recent years. Murine Mincle is responsible for the commensal-detecting function of the intestinal Peyer’s patches to regulate interleukin-17 (IL-17) and interleukin-22 (IL-22) production from innate lymphoid cells (ILC) and T cells, ensure the production of many intestinal antimicrobial proteins (e.g., RegIIIγ, IgA), and eventually restrict aberrant microbial translocation (99). Besides, another study revealed the importance of a CX3CR1^+^ gut-resident mononuclear phagocytes (MNPs), which are a subset of phagocytes equipped with a high expression of genes involved in fungal recognition (including Mincle, Dectin-1, and Dectin-2). It function as a novel mediator of the interactions between fungal community and host intestinal immunity in a Syk-dependent manner (100). Consistent with the observation that CX3CR1^+^ MNPs-depleted mice exhibit gut microbiota alteration and severe DSS-induced colitis, the CX3CR1 gene missense mutation in CD patients is associated with impaired antifungal antibody responses (100). Furthermore, compared with CD remission patients and health controls, the serum levels of SAP130, a classic DAMP of Mincle, is significantly increased in active CD patients (101). Meanwhile, both SAP130 and Mincle in colon tissues of active CD patients is upregulated, which provide a promising biomarker to monitor the severity of CD and the clinical efficacy of treatment (101).

Then a latest study further illustrates a direct link between Mincle signaling and intestinal inflammation (102). Biopsies from CD patients, DSS-induced mouse colitis model and TNBS-induced mouse colitis model consistently suggest that increased expression of Mincle is correlated to inflammation severity. The immune cells that predominantly express Mincle during inflammation are proven to be macrophages in intestinal lamina propria. After damaged tissues produce SAP130, the activated Mincle signaling in macrophage not only leads to inflammatory cytokines release through macrophage pyroptosis, but also recruits neutrophils through the production of chemokines (e.g., Cxcl2 and IL-8). Deficiency of Mincle or inhibition of its downstream Syk both ameliorate experimental colitis by restricting macrophage pyroptosis and limiting the release of proinflammatory cytokines and chemokines. Conversely, when adding a synthetic analog of TDM to bind and activate Mincle, the disease is deteriorated, highlighting that the Mincle/Syk axis is a novel target for the therapeutics of CD. In addition to anti-Mincle neutralizing antibody which has been shown to improve experimental colitis, effective agent to inhibit Mincle hasn’t been developed till now, which is worth exploring.

### Dectin-3

Macrophage C-type lectin(MCL, Dectin-3) is a transmembrane CLR expressed on macrophages (103), monocytes, neutrophils and DCs. Tumor necrosis factor (TNF) can upregulate Dectin-3, Mincle, and Dectin-2 in macrophages (104). Dectin-3 is also linked to FcRγ (35), and acts as an activating receptor capable of initiating immune signaling pathway *via* Syk, CARD9 and NF-κB, which is important for antimicrobial defense, phagocytosis, cytokine expression, and respiratory burst (105, 106).

Dectin-3 can selectively bind α-mannans derived from diverse fungi (e.g., *Candida albicans*, *Paracoccidioides brasiliensis*) and lead to NF-κB activation and defense response (107, 108). Dectin-3 also recognizes TDM of *Mycobacterium tuberculosis* (**Table 1**) and induces Mincle expression upon TDM stimulation (35). Dectin-3 can cooperate with Dectin-2 to form a heterodimeric PRR, which binds α-mannans more potently comparted to their homodimers, thus inducing powerful inflammatory response against fungi (107). Besides, Dectin-3 can also form heterodimer with Mincle (109), implying that various heterodimers and homodimers formed by distinct CLRs greatly elevate the sensitivity and expand the diversity required for monitoring a wide range of microbial infections (107).

In addition to deteriorating experimental autoimmune encephalomyelitis (110) and systemic lupus erythematosus (111), Dectin-3 was initially found a limited role in regulating intestinal immunity (112), while later research revealed that Dectin-3 was also significant in intestinal homeostasis through interplaying with commensal fungi. The deficiency of Dectin-3 leads to more susceptibility to DSS-induced mouse colitis, with an obviously increase of specific fungal burden and microbial translocation, especially a common commensal *Candida tropicalis (* 113). Administration with *Candida tropicalis* only worsens colitis in Dectin-3-deficient mice, because Dectin-3 deficiency impedes NF-κB activation, which leads to defects in cytokine production, epithelial restitution, macrophage phagocytosis during fungal invasion (113). When treated with antifungal therapy, intestinal inflammation can be effectively suppressed in Dectin-3 deficient mice (113). Furthermore, a latest study found that a negative regulatory factor downstream of Dectin-2 and Dectin-3 signaling pathways, E3 ubiquitin ligase c-Cbl, is also involved in regulating intestinal fungi-induced inflammation (114). Commensal fungi-derived mannans can facilitate the transcription of gene *il10* and the expression of anti-inflammatory cytokine to decrease susceptibility to colitis in wild-type mice through activating Dectin-2, Dectin-3 and their downstream c-Cbl in DCs (114). However, c-Cbl deficiency leads to activation of noncanonical NF-κB subunit RelB during mannan stimulation, which suppresses canonical NF-κB subunit p65-mediated *il10* transcription, thus eventually exacerbating DSS-induced mouse colitis (114).

### DCIR

Dendritic cell (DC) immunoreceptor (DCIR) is expressed on DCs, monocytes, macrophages, granulocytes, NK cells, B cells and activated T cells (**Table 1**) (38, 115, 116), and contains a single CRD and an intracellular ITIM (116). The expression of DCIR on DCs relies on their origin and developmental stage, for example, the down-regulation of DCIR is associated with stimuli that induce DCs maturation (e.g., LPS, TNF-α) (116).DCIR is mannose/fucose-binding lectin, which can interplay with both microbial and endogenous ligands (117). Interestingly, DCIR also interacts with HIV-1 and contributes to viral infection (118). The interaction between DCIR and ligands was substantially affected by the glycosylation of the CRD domain in DCIR (117, 119). The special cytoplasmic ITIM of DCIR is responsible for transducing immunoregulatory signals *via* recruiting SHP-1 and SHP-2 (27). DCIR signaling not only suppresses the TLR8-induced production of IL-12 and TNF-α in myeloid DCs but also inhibits the TLR9-induced expression of IFN-α in plasmacytoid DCs (120, 121). Besides, DCIR suppresses the function of CD8α^−^ conventional DCs (cDCs), and the deficiency of DCIR results in TLR-mediated hyperinflammation and reinforced T cell responses against microbes (122).

DCIR may play a different and even opposite role in different inflammatory processes. In addition to ameliorating autoimmune arthritis (123) and exacerbating brain inflammation (124), DCIR also take part in the pathogenesis of IBD. Compared to wild-type mice, there is only a little exacerbation of intestinal inflammation in DCIR-deficient mice in an early study using DSS-induced murine colitis model, hinting an insignificant role of DCIR in intestinal homeostasis (112). However, another murine experiment shows that DCIR1 deficiency is associated to decreased accumulation of neutrophils that shows destructive characteristics and reduced neutrophil-recruiting chemokine MIP-2 in DSS-induced colitis (125), and massive infiltration of neutrophils is known to be associated with the pathogenesis of ulcerative colitis (126) and DSS-induced mice colitis (127). In summary, current studies on the relationship between DCIR and colitis are not sufficient, and the results are not convincing, so further exploration is needed in this field.

### DC-SIGN

Another well-studied CLRs, Dendritic cell-specific intercellular adhesion molecule-3-grabbing non-integrin (DC-SIGN) is mainly expressed on human DCs and macrophages. DC-SIGN on DCs and ICAM-3 on T cells mediate intercellular contact and stabilize their contact zone, which is important for T cell immune response (31). Unlike the CLRs described above, DC-SIGN don’t contain evident ITAM or ITIM domains, but the stimulation of DC-SIGN by various pathogens can regulate TLR signaling *via* serine and threonine kinase Raf-1 and resulting in subsequent acetylation of the NF-κB subunit p65 (128). With prolonged NF-κB transcriptional activity and elevated *il10* transcription rate, acetylation of p65 facilitate anti-inflammatory cytokine responses (128).

DC-SIGN is Ca^2+^ dependent fucose/mannose binding lectin (**Table 1**), and these carbohydrate structures are abundantly expressed by many exogenous pathogens and endogenous ligands. Exogenous ligands consist of virus, helminths, bacteria and fungi, such as HIV-1 (129), *Schistosoma mansoni (* 130), *Mycobacterium tuberculosis (* 130), *Helicobacter pylori (* 130), *Pseudomonas aeruginosa (* 131), and *Candida albicans (* 132). DC-SIGN is critical for DCs responses against pathogens, with different pathogens interacting with DC-SIGN to modulate different TLRs signaling (128). Interestingly, the recognition of mannans by host has been demonstrated to exhibit quite a few functional redundancies with various PRRs involved, such as TLR2, TLR4, Mincle, Dectin-3, Dectin-1 and DC-SIGN. While different *Candida* spp. rely on different kinds of mannan-detecting PRRs, because of disparate mannan composition in fungal cell wall (133).

In addition, various endogenous ligands, including ICAM-3 (31), ICAM-2 (134), Mac-1 (135, 136), Mac-2BP (137), MSPL/TMPRSS13 (138), CEA (139, 140) and CEACAM1 (136), have been reported to take part in intercellular recognition and interaction *via* DC-SIGN (**Table 1**). Interestingly, oncogenesis causes altered glycosylation in tumor cells and elevates the presence of DC-SIGN-binding carbohydrate on tumor associated antigens, such as carcinoembryonic antigen (CEA) (139, 140). Although many of these ligands of DC-SIGN also bind DCIR, there is DC-SIGN-specific ligands, such as *Candida albicans* and glycoproteins on certain cancer cells (117).

Several animal experiments have discovered that mouse homologs of human DC-SIGN take part in the development of colitis. After the administration with DSS, SIGN-R1-deficient mice are more resistant to colitis and exhibit less severe intestine injury and lower expressions of proinflammatory cytokines than wild-type mice, which is associated with defective macrophage responsiveness to commensal lipopolysaccharide (LPS) stimulation (141). Furthermore, SIGN-R1 and TLR4 acts synergistically to regulate intestinal inflammation (141). In contrast, another murine homolog SIGN-R3 can recognize carbohydrate ligands on commensal fungi, and the SIGN-R3-deficient mice exhibit more severe colitis symptoms (such as weight loss and diarrhea) with increased TNF-α production in colon compared to wild-type mice (142). Besides, it’s worth noting that SIGN-R3 contains hemITAM signal motif, while both DC-SIGN and SIGN-R1 belong to ITAM-ITIM-independent CLRs (27). Interestingly, a recent finding shows that the interaction between surface layer protein A (SlpA) in food-grade probiotics *Lactobacillus acidophilus* and murine SIGN-R3 protects intestinal mucosal barrier, prevents dysbiosis, promotes colonic regulatory signaling and finally helps to mitigate experimental colitis, while these protective roles don’t exist in SIGN-R3-deficient mice (143).

## The Role of CLRs in Intestinal Carcinogenesis

Inflammation can facilitating neoplastic progression by contributing to multiple hallmark capabilities of tumor (144). To be specific, inflammation can supply various bioactive molecules to the tumor microenvironment (TME), including proangiogenic factors, growth factors, survival factors, mutagenic reactive oxygen species, epithelial-mesenchymal transition (EMT) activation signals, and extracellular matrix-modifying enzymes that facilitate metastasis. Furthermore, as an essential downstream signal of numerous CLRs, NF-κB also functions as an important bridge between inflammation and carcinogenesis (145).

The close relationship between inflammation and cancer is particularly prominent in colorectal cancer (CRC). As the third most common malignancy, CRC causes a vast amount of cancer-related death around the world (146). Chronic colonic inflammation in UC or CD patients is a well-recognized risk factor for colon carcinogenesis (147, 148). Unlike sporadic CRC that progresses based on precancerous lesion (i.e. colorectal adenomas), colitis-associated cancer (CAC) usually progresses in an order of indefinite dysplasia, low-grade dysplasia, high-grade dysplasia, and finally carcinoma (148). Many pro-inflammatory cells and molecules in chronic inflammation have also been confirmed to influence the progression of CAC. Besides, inflammation is also involved in sporadic CRC though less understood (149). In contrast to normal colon, inflamed colon exhibits higher mutation frequency of many genes(such as tumor suppressor gene p53) under the influence of reactive oxygen and nitrogen species even before there is any evident dysplasia in tissues (150). The risk of CRC is positively related to prolonged colitis duration, severity of inflammation, extensive anatomic extent of colitis and other inflammatory comorbidity (especially primary sclerosing cholangitis), for example, CRC rarely happens in patients whose duration of IBD is less than 7 years, but the CRC risk increases year by year with the prolonged IBD duration after diagnosis (148). Whereas the risk of CRC decreases when IBD patients take anti-inflammatory agents (such as steroids) (148). A better knowledge of the pathogenesis of CRC or CAC is of great significance to define preventive, diagnostic, and prognostic protocols.

Because the intestinal fungal dysbiosis is closely involved in the pathogenesis of IBD, fungi may be also involved in the development of CRC. More exactly, the pathogenesis of CRC including CAC is closely associated with the sophisticated interplay between intestinal immune system and flora (151–153). By comparing fecal fungal microbiota (i.e. mycobiota) of colon polyp patients, CRC patients, and healthy controls, obvious fungal dysbiosis is observed in polyp and CRC groups, including decreased fungal diversity, increased *Ascomycota*/*Basidiomycota* ratio, and an increased abundance of opportunistic pathogenic fungi *Trichosporon* and *Malassezia*, implying a role of fungi in CRC (153). It should be noted that fecal microbiota is not exactly equivalent to mucosal microbiota (154). Then deep sequencing characterized the fungal profile using biopsies of adenomas and paired adjacent tissues, but healthy biopsy samples are unavailable in this study because of ethical issues (155). The results revealed that three fungal phyla, *Ascomycota*, *Glomeromycota* and *Basidiomycota* were dominant in all biopsy samples, and two rare phyla, *Chytridiomycota* and *Neocallimastigomycota* are present in partial samples with relative abundance less than 1% (155). Although the fungal diversity was lower in adenomas than in adjacent tissues, two opportunistic pathogenic fungal genera, *Phoma* and *Candida*, were abundant both in adenomas and adjacent tissues (155). More importantly, the fungal dysbiosis is significantly related to the size and stage of adenomas (i.e. advanced and non-advanced) (155). Likewise, a similar tendency of fungal alteration is also present in the IBD study (51, 52, 56). Interestingly, by collecting fecal and mucosal samples from CRC patients, polyps patients and healthy controls, other researchers found that bacterial microbiota dysbiosis existed not only in cancerous tissues but also in noncancerous tissues, and exhibits differences between distal CRC and proximal CRC, though there is still some confounding factor behind this result (154). By transferring human fecal microbiota to germ-free mice, a recent study found CRC-associated microbiota dysbiosis contributed to oncogenic epigenetic alterations by inducing more hypermethylated genes in murine colonic mucosa, and cumulative methylation index is an independent risk factor for CRC (156). In addition, many mycotoxins and fungal metabolites also directly promote carcinogenesis, for example, *Candida albicans* can product nitrosamine, acetaldehyde and candidalysin (157). Furthermore, a latest study has just found that not only bacterial microbiota (e.g., *Fusobacterium*) but also fungal microbiota can affect chemotherapy resistance (158). Both in CRC patients and in animal experiments, the burden of *Candida tropicalis* is significantly elevated, which can increase the resistance to oxaliplatin treatment *via* facilitating the production of lactate and inhibiting the mismatch repair system (158).

Above all, both intestinal inflammation and fungal disorders have been confirmed important roles in the pathogenesis of CRC. As a key mediator of fungal recognition and immune response, CLRs also take part in the progression and development of CRC (**Figure 1**).

### Dectin-3

The impact of Dectin-3 on CAC development has just been revealed. After administration with azoxymethane (AOM) and DSS, Dectin-3-deficient mice showed exacerbated CAC tumorigenesis, increased *Candida albicans* burden and impaired immune responses compared to wild type mice (159). Furthermore, germ-free mice that is colonized with *C. albicans* shows more severe colitis and CAC during AOM-DSS administration, and treatment with antifungal fluconazole ameliorates chemically induced-CAC in Dectin-3-deficient mice (159). By carrying out a variety of experiments such as fecal microbiota transplantation, the putative mechanism behind this phenomenon is as follows (159). The impaired fungicidal abilities of Dectin-3 deficient macrophages lead to intestinal fungal dysbiosis, especially increased *C. albicans* burden, which triggers glycolysis in macrophage to produce IL-7, then promotes innate lymphoid cells ILC3 to produce IL-22 under the control of IL-7, *Stat3* and *AhR*, and IL-22-induced p-STAT3 in intestinal epithelial cells eventually promotes CAC tumorigenesis. Consistently, a similar mycobiota/Dectin-3/IL-22 regulatory axis exists in human CRC patients. Patients with more advanced CRC tumors show significantly lower Dectin-3 expressions, and CRC patients with higher fecal fungal burden exhibit lower Dectin-3 expression, higher levels of IL-22 in tumor tissues and poorer disease-free survival and overall survival (159). What’s more, Dectin-3 and Dectin-2 can also act cooperatively to limit liver metastasis by promoting Kupffer cells to phagocytize cancer cells (160).

### DC-SIGN

Long time ago, researchers have realized that DCs recognize tumor-specific antigen on CRC tumor cells through DC-SIGN to affect effective antitumor responses thus helping tumor escape immunosurveillance (139, 140, 161). Malignant transformation alters the glycosylation process and causes increased levels of Lewis X and Lewis Y on tumor-specific CEA in intestinal epithelial cells(IECs), which can be selectively recognized by DC-SIGN to mediate the interaction between DCs and tumor cells, while normal IECs contains low levels of Lewis antigens on CEA thus avoiding DC-SIGN binding (139). Besides, primary CRC tissues from some patients express a novel DC-SIGN ligand, Mac-2-binding protein (Mac-2BP), which also contained special glycosylated structures (137). The interactions between DC-SIGN and CRC-specific glycosylation inhibit functional maturation and differentiation of Monocyte-Derived Dendritic Cells (MoDCs) and enhance anti-inflammatory cytokine secretions (e.g., IL-6 and IL-10), which might supply a tolerogenic microenvironment for CRC (137, 161). Interestingly, except in a glycan-dependent manner, DC-SIGN can interact with Type II Serine Protease MSPL/TMPRSS13 on CRC cells in a glycan-independent manner (138). A case-control study found single nucleotide polymorphisms (SNPs) in DC-SIGN were associated with CRC risk (162). Furthermore, CRC patients exhibit decreased serum levels of soluble DC-SIGN, thus meaning a diagnostic significance (163). Additionally, compared to normal colon tissues, the levels of DC-SIGN are higher in the tumor stroma and the invasive margin of CRC tissues, and higher levels of DC-SIGN in CRC tissues are correlated with lower serum levels of DC-SIGN from the same patient (163). Then a systematic review also proves that DC-SIGN/DC-SIGNR is one of the most promising circulating markers for CRC diagnosis (164).

A recent study has shed more light on the mechanism of how DC-SIGN affects CRC. Both infiltrated DCs and cancer cells in colon tumor tissues can express DC-SIGN, whose overexpression was closely linked with more aggressive and invasive tumor, worse prognosis and shorter metastasis-free survival in CRC patients (165). The mechanism is that DC-SIGN activation promotes the transcription of MMP-9 and VEGF *via* PI3K/Akt/β-catenin pathway and suppresses the transcription of miR-185 *via* β-catenin/TCF1/LEF1 pathway, which eventually promotes metastasis of CRC (165). The DC-SIGN signaling pathway in metastatic CRC reveals a new pathogenesis of CRC and provides new targets for blocking the invasion and metastasis of CRC. Another study demonstrated that CRC craftily take advantage of the overexpression of complex branched N-glycans to help tumor cells escape immune recognition and construct immunosuppressive microenvironment with inhibited production of IFN-γ and increased frequency of Treg (166). Intriguingly, the removal of this branched N-glycans on CRC cells could expose immunogenic glycan epitopes to enhance recognition by immune cells *via* DC-SIGN and potentiate an effective antitumor immune response (166). Besides, another member of DC-SIGN family, DC-SIGNR, could promote colon carcinoma hepatic metastasis (167).

### LOX-1

The lectin-like oxidized low-density lipoprotein receptor-1 (LOX-1) is expressed by vascular endothelial cells (168), vascular smooth muscle cells, cardiomyocytes, adipocytes, platelets, monocytes, macrophages, DCs, B cells, chondrocytes (169) and intestinal cells (170) (**Table 1**), and function as a scavenger receptor (171). The low expression of LOX-1 under physiological conditions can be up-regulated by many diseases-related stimuli, such as cytokines (e.g., TGF-β1 (172), TNF-α (173)), oxidized low-density lipoprotein (oxLDL) (174, 175), angiotensin II (176), endothelin (177), asymmetric dimethylarginine (178). There is no known ITAM or ITIM in LOX-1 cytoplasmic tail, but LOX-1 can also mediate NF-κB pathway, thus implying LOX-1 utilizes other molecules for intracellular signaling transduction, which needs further elucidation (27).

LOX-1 is responsible for the recognition and adhesion of exogenous Gram-positive and Gram-negative bacteria, such as *Staphylococcus aureus* and *Escherichia coli (* 179). Furthermore, Ox-LDL can bind LOX-1 and there is considerable evidence for their pathogenic role in atherosclerosis. Based on LOX-1 mediated MAPK/NF-κB pathway activation, Ox-LDL elevates the levels of LOX-1, promotes the maturation and differentiation of DCs and induces a proinflammatory cytokine profile (174, 175). Heat shock proteins(HSP) also bind to LOX-1, and anti-LOX-1 antibody impedes such ligation thus inhibiting HSP-induced antigen cross-presentation (40). In addition, oxidized high-density lipoprotein (ox-HDL), phosphatidylserine (PS), apoptotic bodies, advanced glycation end-products (AGEs), and platelets all acts as LOX-1 ligands (**Table 1**).

LOX-1 has attracted much attention in the cardiovascular field. Altered expression of LOX-1 is linked to risks of various metabolic diseases, for example, atherosclerosis, hyperlipidemia, diabetes, obesity (180). Besides, soluble LOX-1(sLOX-1) can be released to serum after proteolytic cleavage, which acts as a promising non-invasive biomarker for many diseases such as type 2 diabetes mellitus (181). Uniquely, LOX-1 signaling also takes part in humoral responses by triggering DC-mediated class-switched B cell to promote antibody responses and changing expressions of chemokines to facilitate B cell migration (182).

More importantly, there is a close link between metabolic dysfunction and malignant tumor with LOX-1 involved in. The prevalence of CRC is higher in patients with coronary artery disease (183) and treatment with statins, an effective lipid-lowering agent, can efficiently reduce the risk of CRC (184). Besides, increased human serum ox-LDL levels not only contribute to atherogenesis, but also correlate to increased risk of CRC (185, 186). As proven by experiments using developmentally diverse cancer cell lines, LOX-1 is crucial for maintaining the growth and transformation of tumor through NF-κB-mediated inflammatory and hypoxia responses (187). Ox-LDL binding to LOX-1 promotes the accumulation of reactive oxygen species (ROS), which contributes to the development and progression of various neoplasia, including CRC tissues (185, 188). Above all, LOX-1 is a crucial molecular bridge to connect cancers with various metabolic diseases.

More direct evidence suggests that human CRC tissues produce excessive ox-LDL and strongly upregulate LOX-1, and the overexpression of LOX-1 correlates to highly aggressive and metastatic human CRC, while metastasis and recurrence are leading causes of CRC mortality (189). The *in vitro* knockdown of LOX-1 in CRC cells impairs proliferation rate, hinders the maintenance of tumorigenicity and influences the presence of peculiar volatile organic compounds (VOCs) (189). Subsequent study using two different xenografting procedures in mice (subcutaneous and endovenous) further revealed that LOX-1 silencing affects not only the engraftment of the tumor but also the development of metastasis, where angiogenesis takes a crucial role (190). And LOX-1 also affects gene transcription through epigenetic regulation such as different histone acetylation pattern (190). The latest study that uses human CRC tissues and CRC xenograft mouse model also found the glycolytic metabolism and chemoresistance of CRC tissues were regulated *via* the upregulation of LOX-1/c-MYC/SULT2B1 axis, and the knockdown of LOX-1 downregulated SULT2B1 *via* c-MYC thus repressing glycolytic metabolism to inhibit the proliferation and chemoresistance of CRC (191). Consistent with these investigations, another recent study reported for the first time that higher serum LOX-1 levels of CRC patients determine poorer overall survival and worse prognosis (192). Serum LOX-1 actually represents an independent prognostic factor and positively correlates with many inflammatory factors, at the same time, patients with high LOX-1 expression in CRC tumor tissues also showed poor prognosis (192). LOX-1 has also been identified as a crucial player in immunosuppression in tumor microenvironment. Neutrophils with high expression of LOX-1 shows similar characteristics of myeloid-derived suppressor cell (MDSC) and exerts powerful immunosuppressive effects such as the inhibition of T cell proliferation, which contributes to the progression and recurrence of glioblastoma (193, 194).

Intriguingly, another recent study found that lower levels of LOX-1 and CD8^+^ cytotoxic T lymphocyte (CTL) in tumor stroma were related to worse prognosis in CRC patients (195). The researchers speculated that the reasons for this phenomenon is that the CRC stromal cells that express LOX-1 were mostly CD163^+^ M2 macrophages, whose infiltration in stroma was beneficial for CRC prognosis while harmful for several other cancers (195). Whether LOX-1 expression influences the quantity and quality of M2 macrophages in CRC microenvironment thus influencing the efficacy of anti-tumor immunity needs further evidences to elucidate.

In summary, LOX-1 not only provides an environment that facilitates tumor progression and invasion, but also mediates immunosuppressive signals to help tumor cells escape from immunosurveillance. Although there are still problems that have not been fully clarified, the current researches point out the potential to utilize LOX-1 as therapeutic target to inhibit CRC tissues growth and metastasis and repress chemoresistance. In addition, the identification and characterization of peculiar VOCs that is induced by LOX-1 may provide a simple, convenient and non-invasive biomarker for CRC diagnosis and monitoring.

### Other CLRs

Although the role of Mincle in the relationship between intestinal fungal dysregulation and CRC is unclear, the ligands of Mincle hold great potential for the development of new cancer vaccine, such as promoting the lysis of mouse CRC cells (196) and enhancing the maturation and migration of DCs to trigger anti-tumor effect in CRC (197). As for Dectin-1, a recent study found that the significantly increased *Candida albicans* in the guts of CRC patients can induce the proliferation of the human intestinal epithelial cells through the Dectin-1 mediated Wnt/β-catenin signaling thus contributing to CRC development (152). But the precise effect of Dectin-1 on CRC is largely unsure.

## The Role of Downstream Adaptor CARD-9 in Intestinal Inflammation and Carcinogenesis

Caspase recruitment domain 9 (CARD9), a caspase recruitment domain-containing signaling protein, functions as a central adaptor molecule to transduce the signaling of ITAM-coupled or hemITAM-bearing CLRs (e.g., Dectin-1, Dectin-2, and Mincle) in the immune response to fungi (198, 199). CARD9 not only mediates CLRs signaling by coupling the activation of Syk to the regulation of NF-κB pathway, but also widely take part in the integration of signals downstream of other PRRs, such as NLRs and TLRs (198, 199). CARD9 signaling molecule is a major player in both innate immunity and adaptive immunity to react against various pathogens properly, including fungi, bacteria, and viruses. Therefore, CARD9 is associated with many inflammatory disorders. For example, human CARD9 deficiency contributes to chronic mucocutaneous candidiasis (200) and invasive *Candida* infections in the central nervous system or digestive tract (e.g., meningoencephalitis, colitis) (201).

CARD9 is a key downstream regulatory molecule to maintain the intestinal homeostasis *via* regulating host immune system and the intestinal flora. Many genome-wide association studies all found that CARD9 polymorphism was strongly related to CD and UC (202–204). Experiments on mice showed that CARD9-null mice had difficulty in recovering from epithelial damage with impaired production of cytokines including IL6, IL-17 and IL-22 when treated with DSS (205). And CARD9 deficiency was associated with suppressed Th17 and innate lymphoid cells (ILCs) in murine colon during *Citrobacter rodentium* infection, suggesting that CARD9 participates in an effective defense response against *C. Rodentium (* 205). In addition, CARD9-null mice showed more susceptibility to colitis companied by strongly elevated fungal burdens and altered intestinal fungal composition (e.g., increased *Candida tropicalis*), which is partially due to impaired fungicidal functions of CARD9-null macrophages, thus implying an inability to accurately control intestinal fungal community is involved in the deteriorated colitis of CARD9-null mice (205–207). What is more, CARD9 gene also affects intestinal inflammation through modulating the production of microbial metabolites. Transferring the intestinal flora from CARD9-null mice to wild-type germ-free mice is proven to promote colitis and reduce IL-22 production, because the microbiota in CARD9-null mice is unable to metabolize tryptophan into the ligands of aryl hydrocarbon receptor (AHR) (207). AHR is important for inducing IL-22 production (208), a critical cytokine for the maintenance of intestinal homeostasis (209). Therefore, supplement with tryptophan-metabolizing *Lactobacillus* strains or treatment with AHR agonist is effective to attenuate intestinal inflammation (207). Consistently, both CD and UC patients shows reduced AHR ligands and defective AHR activation in their microbiota, especially for patients with CARD9 risk alleles (207). Furthermore, IBD patients with CARD9 risk alleles shows more abundant *Malassezia restricta* in the gut, which usually exists as a skin commensal fungus (210). In germ-free mice with or without bacteria colonization, increased levels of *M. restricta* alone are enough to directly exacerbate colitis, because *M. restricta* can elicit a strong proinflammatory response in a CARD9-dependent manner, implying the potential to utilize specific commensal fungi as a therapeutic target for IBD (210).

Syk-CARD9 signaling mediates a protective role in the interplay between fungal microbiota and CAC. CARD9-null mice significantly altered microbial profile, which is associated to CAC (206, 211). Upon administration with AOM-DSS, CARD9-null mice are proven to be more susceptible to CAC, whose mechanism is that defective fungicidal functions of Card9-null macrophages lead to fungal dysbiosis (especially increased *Candida tropicalis*), and *C. tropicalis* increases the number of intestinal MDSC and activates the function of MDSC (206). Antifungal treatment (fluconazole) can ameliorate CAC in CARD9-null mice together with decreased accumulation of MDSC (206). Similarly, CRC patients shows higher proportion of *C. tropicalis* and patients with higher levels of fecal fungal burden exhibit increased MDSCs in their blood and colon tissues (206). Likewise, mouse Lewis lung cancer model revealed a noncanonical CARD9/NF-κB/IDO pathway, which can limit tumor progression through inhibiting the immunosuppressive capacity of MDSCs (212). In addition to MDSCs accumulation, the deletion of CARD9 or Syk also increases susceptibility to colitis and CAC in mouse model because of another protective mechanism, microbiota/Syk/CARD9/IL-18 axis, where commensal fungi promote the activation of inflammasome and the maturation of IL-18 in a CARD9-dependent manner to influence the restitution and maintenance of intestinal epithelial barrier and the production of IFN-γ (211). Treatment with amphotericin B (AmpB) to deplete commensal fungi in wild type mice can exacerbated CAC while supplementation with IL-18 or wildtype myeloid cells in CARD9-null mice can ameliorate CAC (211).

However, CARD9 has also been shown promoting tumor effects in various types of malignant diseases, including CAC. CARD9 can promote CAC *via* CARD9/IL-1β/IL-22 axis and the IL-22 production can promote tumorigenesis *via* epithelial STAT3 activation (213). Furthermore, another study also found that CARD9 promoted liver metastasis of CRC tissues through metastasis-associated macrophage polarization *via* NF-κB pathway activation (214). Interestingly, using the mouse model of human familial adenomatous polyposis (FAP), CARD9 is revealed to exacerbate intestinal neoplasia in a sex-biased manner where male mice showed reduced viability, more tumor burden and more immune cells infiltration, implying gender differences in human CRC may involve CARD9-dependent inflammation (215).

## Prospectives

Fungal dysbiosis and related immune responses contribute to the pathogenesis of IBD and CRC, which is implicated to be mediated by CLRs, especially Dectin-1, Mincle, Dectin-3 and their downstream adaptor protein CARD9. However, the downstream signaling pathways of many CLRs and detailed mechanisms of aberrant CLRs signaling affecting IBD or CRC are complex and still unclear. In particular, there are still many discrepancies and even contradictions in the current research results on the role of CLRs in IBD and CRC. There are several possible reasons for this paradox, for example, different diet in experimental animals and undiscovered crosstalk within distinct PRRs. To be specific, different diets may affect intestinal inflammation in mice by affecting their gut microbiota composition (94). And CLRs can interfere with other PRRs-mediated signal transduction, such as TLR4, so simultaneous activation of multiple PRRs may produce different effects (41). In addition, experimental dose, mouse strain, sex bias and the interaction between intestinal fungi and bacteria may also influence experimental results. All these factors need to be considered in future studies.

The improvement of next generation sequencing (NGS) and third generation sequencing (TGS) technology paves the way to further investigate intestinal microbiota. A boom in the study of intestinal bacteria is facilitated by 16S rRNA sequencing, and in the past few years, a lot of emerging fungal sequencing studies are based on internal transcribed spacer (ITS) region and 18S rRNA (216). The combination of ITS and18S rRNA sequencing could provide a more comprehensive characterization of intestinal fungi (216). It should be noted that intestinal fungal studies that utilize metagenomics and metatranscriptomic analysis are limited till now (217). In addition, as an important turning point in microbiota research, newly-developed culture-dependent strategies, such as high-throughput culturomics combined with matrix-assisted laser desorption ionization-time of flight mass spectrometry (MALDI-TOF MS) and ITS sequencing, make up for the deficiency of sequencing research and has been applied in fungal microbiota research (218). Other cutting-edge technologies can also be applied to the study of intestinal flora, for example, genome-wide CRISPR-Cas9 screen helps researchers to find the receptors for toxins of *Enterococcus* (219).

These latest technology and research paradigms help to elucidate the molecular mechanism of intestinal microbiota-inflammation-cancer axis. Newly discovered biomarkers and targets in this axis could be utilized to develop innovative vaccines and medicines, so as to approach more efficient and more individualized treatment. For example, through taking advantage of the adjuvant capacity of Mincle agonist, conjugating model antigen with Mincle agonist is a new pathway to develop cancer vaccines, which has been proven to provoke strong anti-tumor immune responses in mice (196). Besides, a new therapeutic agent, cationic liposome TDM, shows anti-tumor effect in mouse tumor models in a Mincle-dependent manner (197). Given the powerful and diverse role of CLRs in modulating innate and adaptive immune responses in IBD and CAC, such as the role of Dectin-1 in Treg amplification and the role of DC-SIGN in tumor metastasis, CLRs is undoubtedly very promising and worth-exploring therapeutic targets.

## Author Contributions

ML: Writing - Original Draft & Manuscript Submission. RZ: Writing- Original Draft, Review & Editing. JL: Supervision, Resources, Funding Acquisition, Writing - Review & Editing. JNL: Supervision, Resources, Funding Acquisition, Writing - Review & Editing. All authors contributed to the article and approved the submitted version.

## Funding

Natural Science Foundation of China (Grant Nos. 81730016 and 81900483), CAMS Innovation Fund for Medical Sciences (CIFMS2021-I2M-C&T-A-001).

## Conflict of Interest

The authors declare that the research was conducted in the absence of any commercial or financial relationships that could be construed as a potential conflict of interest.

## Publisher’s Note

All claims expressed in this article are solely those of the authors and do not necessarily represent those of their affiliated organizations, or those of the publisher, the editors and the reviewers. Any product that may be evaluated in this article, or claim that may be made by its manufacturer, is not guaranteed or endorsed by the publisher.
